# Achieved systolic blood pressure in older people: a systematic review and meta-analysis

**DOI:** 10.1186/s12877-017-0672-4

**Published:** 2017-12-05

**Authors:** Aline A. I. Moraes, Cristina P. Baena, Taulant Muka, Arjola Bano, Adriana Buitrago-Lopez, Ana Zazula, Bruna O. Erbano, Nicolle A. Schio, Murilo H. Guedes, Wichor M. Bramer, Oscar H. Franco, José Rocha Faria-Neto

**Affiliations:** 1 Pós Graduação de Ciências da Saúde, Pontifícia Universidade Católica do Paraná, Rua Imaculada Conceição, 1155 – Parque Tecnológico 06, Curitiba, Paraná CEP: 80.215-901 Brazil; 2 Department of Epidemiology, Erasmus MC, Erasmus MC, University Medical Center Rotterdam Office Na 29-16, PO Box 2040, 3000 CA Rotterdam, The Netherlands; 3 Universidad Javeriana. Pontificia Universidad Javeriana, Carrera 7 No. 40 – 62, Bogotá D.C., Colombia; 4 Escola de Medicina, Pontifícia Universidade Católica do Paraná, Rua Imaculada Conceição, 1155 – Parque Tecnológico 06, Curitiba, Paraná CEP: 80.215-901 Brazil; 5 Escola de Medicina, Faculdade Evangélica do Paraná, Rua Padre Anchieta, 2770, Curitiba, Paraná CEP80730-000 Brazil; 6 Medical Library Erasmus MC, University Medical Center Rotterdam Office Na 29-16, PO Box 2040, 3000 CA Rotterdam, The Netherlands

**Keywords:** Aged, Older people, Blood pressure, Hypertension, Antihypertensive agents, Antihypertensive drugs, Antihypertensives

## Abstract

**Background:**

It remains unclear into which level the systolic blood pressure (SBP) should be lowered in order to provide the best cardiovascular protection among older people. Hypertension guidelines recommendation on attaining SBP levels <150 mmHg in this population is currently based on experts’ opinion. To clarify this issue, we systematically reviewed and quantified available evidence on the impact of achieving different SBP levels <150 mmHg on various adverse outcomes in subjects aged ≥60 years old receiving antihypertensive drug treatment.

**Methods:**

We searched 8 databases to identify randomized controlled trials (RCTs) and post-hoc analyses or subanalyses of RCTs reporting the effects of attaining different SBP levels <150 mmHg on the risk of stroke, acute myocardial infarction, heart failure, cardiovascular mortality and all-cause mortality in participants aged ≥60 years. We performed random-effects meta-analyses stratified by study design.

**Results:**

Eleven studies (> 33,600 participants) were included. Compared with attaining SBP levels ≥140 mmHg, levels of 130 to <140 mmHg were not associated with lower risk of outcomes in the meta-analysis of RCTs, whereas there was an associated reduction of cardiovascular mortality (RR 0.72, 95% CI 0.59–0.88) and all-cause mortality (RR 0.86, 95% CI 0.75–0.99) in the meta-analysis of post-hoc analyses or subanalyses of RCTs. Limited and conflicting data were available for the SBP levels of <130 mmHg and 140 to <150 mmHg.

**Conclusions:**

Among older people, there is suggestive evidence that achieving SBP levels of 130 to <140 mmHg is associated with lower risks of cardiovascular and all-cause mortality. Future trials are required to confirm these findings and to provide additional evidence regarding the <130 and 140 to <150 mmHg SBP levels.

**Electronic supplementary material:**

The online version of this article (10.1186/s12877-017-0672-4) contains supplementary material, which is available to authorized users.

## Background

High blood pressure is one of the leading global risk factors in terms of cardiovascular disease burden [1–3], and its impact on mortality rates increases with age [4]. As the process of population aging develops worldwide, the burden associated with high blood pressure levels is expected to rise [4].

Antihypertensive drugs reduce cardiovascular events and all-cause mortality in older people, as demonstrated by randomized clinical trials (RCTs) comparing treatment with placebo [5–7]. Because in most of these RCTs the mean achieved systolic blood pressure (SBP) in the intervention group was between 140 and 150 mmHg [5, 6], current guidelines recommend attaining SBP levels <150 mmHg during antihypertensive drug treatment among older people [8, 9]. Studies on lower SBP levels yielded conflicting results regarding cardiovascular protection [10–13]. Furthermore, older people have a higher risk of adverse events, particularly concerning kidney failure and symptomatic hypotension, which may further lead into falls and fractures [14]. Therefore, based on experts’ opinion, current hypertension guidelines state that individual tolerability should be considered when recommending SBP < 140 mmHg [8, 9], and it remains unclear into which level the SBP should be lowered in order to provide the best cardiovascular protection without a significant increase in serious adverse events in older people. A comprehensive literature review might help clarify current evidence on the subject.

We aimed to systematically review and quantify available evidence on the impact of achieving different SBP levels <150 mmHg on stroke, acute myocardial infarction (AMI), heart failure (HF), cardiovascular mortality, all-cause mortality and adverse events in subjects aged ≥60 years old receiving antihypertensive drug treatment.

## Methods

### Data sources, search strategy, and eligibility criteria

Eight electronic databases (MEDLINE, EMBASE, Cochrane library, Web-of-science, Lilacs, Scielo, PubMed and Google Scholar) were searched from their inception until July 8th 2016 with no language restriction. Searches combined terms related to the exposure (e.g., antihypertensive drug treatment), cardiovascular outcomes (*e.i*, stroke, AMI, HF, cardiovascular mortality) and all-cause mortality. We also searched for unpublished studies in Clinical Trials and European Medical Agency registries [15–18], asked for experts’ opinion and assessed reference lists of relevant bibliography (review articles, guidelines and original studies identified by the electronic searches) to find other eligible trials. Details of the search strategy are provided in the Additional file 1 and the accordance with PRISMA Statement for reporting Systematic Review and Meta-analysis is described in the Additional file 2.

Inclusion criteria were (i) randomized clinical trials (RCTs), post-hoc or subanalyses of RCTs (p-h/sa of RCTs); (ii) assessing the effects of antihypertensive drug treatment; (iii) achieving different SBP levels or a minimum difference of at least 3 mmHg in SBP between intervention and control groups; and (iv) collecting endpoints for cardiovascular outcomes and all-cause mortality. The 3 mmHg minimum achieved SBP difference between intervention and control groups cut-off was chosen based on previous findings suggesting that a minimum reduction of 4.6 mmHg in SBP is required to prevent a cardiovascular event [19]. The lower cut-off (3 mmHg) was chosen to ensure a conservative approach and has been previously described [20]. Since we aimed to compare the effects of antihypertensive drug treatment targeting different SBP levels, we excluded studies in which all participants on the control group received placebo only or no treatment. We also excluded studies in which: (i) antihypertensive drugs were used for other purposes than lowering blood pressure or in emergency conditions (e.g. angina, arrhythmia, HF or chronic kidney disease); (ii) both intervention and control groups achieved a SBP > 150 mmHg; (iii) final SBP was not described or was not reported for participants ≥60 years old. We followed the 8th Joint age value cut-off (≥ 60 years old) [9].

All titles, abstracts and full texts were assessed independently by two reviewers. Full texts were retrieved for studies that satisfied all selection criteria.

### Data extraction

Two independent authors extracted all relevant data including location, study design, baseline characteristics (mean age, gender, race, hypertension, diabetes, smoking status and previous cerebrovascular or coronary artery disease), follow-up time, class of antihypertensive drugs prescribed, baseline and achieved systolic and diastolic blood pressure, number of participants and events, all strokes (fatal and non-fatal), all AMI (fatal and non-fatal), all HF outcomes (fatal and non-fatal), cardiovascular mortality (according to each study definition), all-cause mortality outcome and adjustments, as well as information on adverse events. For definition of serious adverse events, we considered those adverse events classified by the study as being serious and those defined as kidney failure and fractures. When data on baseline characteristics or adverse events were not reported for participants aged ≥60 years old, we used data from the whole population [13, 21, 22]. When risk estimates were not reported on a RCT, we calculated relative risk (RR), standard error and 95% confidence interval (95% CI) [23] on the basis of the available information from the article [10, 24]. Risk of bias was assessed using the Cochrane Collaboration’s tool [25] for RCTs and the Newcastle-Ottawa Scale [26] for p-h/sa of RCTs.

### Data synthesis and analysis

We classified studies based on the actual cut-offs that the included studies had used. We considered as intervention and control groups those in which achieved SBP were, respectively, lower and higher. These criteria allowed us to compare the following groups: 130 to <140 versus ≥ 140 mmHg, < 130 versus ≥ 130 mmHg, and both groups within the range 140 to <150 mmHg. More detailed information on data synthesis is provided in Additional file 1.

We performed meta-analysis for each SBP level comparison considering RCTs and p-h/sa of RCTs separately and using random effects model. To be able to perform sensitivity analyses and to assure quality, results were pooled when there were at least 3 observations on the outcome available from studies with moderate to low risk of bias. Data from outcomes that did not fulfil criteria for pooling results were reported as part of the systematic review. Hazard ratios were assumed approximate measures of relative risk. Heterogeneity was assessed using the Cochrane χ2 statistic and the I^2^ statistic.

Fixed effect models analyses were conducted as sensitivity analyses and we also provided sensitivity analyses after exclusion of studies on secondary prevention of stroke and coronary heart disease [27], after exclusion of studies in which it was not possible to define whether a fraction of the participants on the control group received only placebo treatment [28], and after exclusion of studies that included participants aged 60 to <65 years old [27]. Publication bias was assessed using Begg funnel plots and the Egger test whenever there were 5 studies or more available for analysis [29].

Baseline data were analysed as weighted means and standard deviation for continuous variables and proportions for categorical variables. All statistical tests were two sided and used a significance level of *p* < 0.05. We used Stata release-14 for all statistical analyses.

## Results

### Study identification and selection

**Fig. 1:**
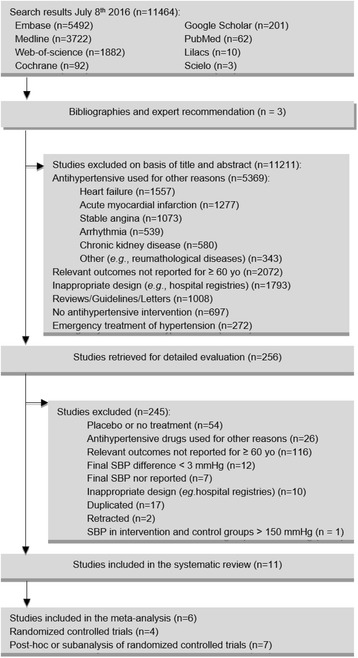
Flow diagram for study selection

The search strategy identified 11,467 citations. Dataset search and the assessment of reference lists of relevant bibliography included 11,464 and 3 citations, respectively. After screening of titles and abstracts, 256 articles were selected for detailed evaluation of their full text. Of those, 11 studies met the inclusion criteria and were included in the review [10–14, 21, 22, 24, 27, 28, 30] (Fig. 1). One study provided independent data for two different age subgroups of participants (65 to 74 and ≥75 years old) [28]. One study compared achieved SBP levels within the 140 to <150 mmHg range [14], 7 studies compared the levels 130 to <140 mmHg with ≥140 mmHg [10, 11, 21, 24, 27, 28, 30], and 3 compared the levels <130 mmHg with ≥130 mmHg [12, 13, 22].

### Characteristics of included studies

**Fig. 2:**
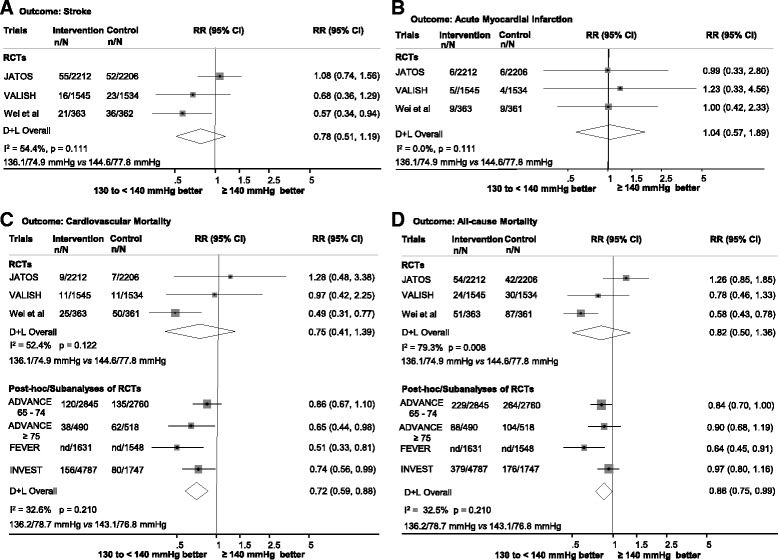
Cardiovascular outcomes and all-cause mortality for SBP 130 to <140 versus ≥ 140 mmHg. Panel **a**: stroke; Panel **b**: acute myocardial infarction; Panel **c**: cardiovascular mortality; Panel **d**: All-cause mortality. Results are stratified by study design. The size of the marker represents the weight of each trial. Weighted average of blood pressure is described for each outcome. SBP, systolic blood pressure; RR, relative risk; 95% CI, 95% confidence interval; RCTs, randomized controlled trials; D + L, DerSimonian-Laird random effect model

Table 1 summarizes the characteristics of included studies and participants. Of the 11 included studies, 4 were RCTs [10, 11, 13, 24] and 7 p-h/sa of RCTs [12, 14, 21, 22, 27, 28, 30]. More than 33,600 participants (mean age of 72 years old) were followed during a mean of 34 months across studies (range 9–55 months). Six studies recruited participants in Europe [12, 14, 22, 27, 28, 30], 4 in America [12–14, 27] and 6 in Asia [10, 11, 14, 21, 24, 28] (Table 1e). Four studies described data on race, with the prevalence of African descendants being <20% in all reports [12, 13, 22, 27]. Eight studies reported data on stroke [10–14, 21, 24, 27], 7 on AMI [10–14, 24, 27], 4 on HF [10, 13, 24, 27], 10 on cardiovascular mortality [10–14, 21, 24, 27, 28, 30], 11 on all-cause mortality [10–14, 21, 22, 24, 27, 28, 30] and 8 on adverse events in older participants (10–14, 24, 27, 28) (Tables 2 and 2e from Additional file 1). Regarding quality assessment, one study was considered to have high risk of bias [30], 6 to have moderate [12, 14, 21, 22, 27, 28], and 4 to have a low risk of bias [10, 11, 13, 24] (Tables 3e and 4e from Additional file 1). At baseline, the mean systolic and diastolic blood pressure of included studies were 158 and 82 mmHg, respectively. The mean achieved systolic and diastolic blood pressure are reported in each pooled outcome in Fig. 2.

**Table 1:** Summary characteristics of included studies and participants according to achieved systolic blood pressure groups

Study name (year)	FU (months)	SBPT^a^ study	P-h/sa of RCTs analysis	N participants		Antihypertensive		Age (SD)	Women (%)	Hypertension (%)	Diabetes (%)	Smoking (%)	Previous coronary disease (%)	Previous cerebrovascular disease (%)
				Intervention	Control	Intervention	Control							
140 to < 150 mmHg														
HOT [14] (2000)	45	No	Yes	nd	nd	Thiazide, BB, CCB or ACEi	Thiazide, BB, CCB or ACEi	70.6 (3.9)	54	100	10	11	13	
130 to < 140 versus ≥ 140 mmHg														
EWPHE [30] (1989)	9	Yes	Yes	120	126	thiazide + triamterene	thiazide + triamterene	71.4 (6.9)	70	100	nd	19	35	
JATOS [10] (2008)	24	Yes	No	2212	2206	CCB	CCB	73.6 (5.2)	61	100	11	13	3	4
VALISH [11] (2010)	33	Yes	No	1545	1534	ARB	ARB	76.1 (4.1)	62	100	13	19	5	6
ADVANCE [28] (2010) 65–74 yo	51	No	Yes	2845	2760	Thiazide + ACEi	Placebo + usual antiHTN drug treatment^c^	68 (nd)	42	61	100	10	11	8
ADVANCE [28] (2010) ≥ 75 yo	51	No	Yes	490	518	Thiazide + ACEi	Placebo + usual antiHTN drug treatment^c^	77 (nd)	40	67	100	6	16	12
FEVER [21] (2011)	40	Yes	Yes	1631	1548	Thiazide + CCB	Thiazide + Placebo	> 65	61^b^	100	12^b^	29^b^	2^b^	10^b^
Wei et al. [24] (2013)	48	Yes	No	363	361	Thiazide, ACEi, BB or CCB	Thiazide, ACEi, BB or CCB	76.5 (4.5)	33	100	23	24	nd	6
INVEST [27] (2014)	24	Yes	Yes	4787	1747	CCB/ACEi or BB/thiazide	CCB/ACEi or BB/thiazide	70.7 (7.3)	55	100	29	42	100	8
< 130 versus ≥ 130 mmHg														
LIFE [22] (2012)	55	Yes	Yes	5704^e^		ARB or BB	ARB or BB	≥ 65	54^b^	100	13^b^	16^b^	16^b^	4^b^
SPS3 [12] (2015)	42	Yes	Yes	248	246	Any	Any	79.9 (3.8)	45	77	27	5	10	100
SPRINT [13, 31] (2016)	37	Yes	No^d^	1317	1319	Any	Any	79.8 (4)	38	nd	0	13^b^	24,5	0

*FU* Follow-up time, *SBPT* Systolic blood pressure target, *P-h/sa of RCTs* Post-hoc analyses and subanalyses of randomized controlled trials, *SD* Standard deviation, *BB*, Beta-blocker, *CCB* Calcium channel blocker, *ACEi* Angiotensin converter enzyme inhibitor, ARB, angiotensin receptor blocker, *antiHTN drug treatment* Antihypertensive drug treatment, *nd* Not described. ^a^Systolic blood pressure target study refers to analysis of different systolic blood pressure targets on cardiovascular outcomes or all-cause mortality. Therefore, studies that are not blood pressure target driven were included based on the criteria of having at least 3 mmHg systolic blood pressure difference between intervention and control group. ^b^ data retrieved from whole population. ^c^ hypertensive participants received antihypertensive treatment; ^d^ SPRINT trial evaluated the elderly population as a prespecified analysis. ^e^ data was combined from intervention and control groups

**Table 2:** Baseline and achieved blood pressure levels and associated cardiovascular outcomes and all-cause mortality

Study name (year)	Baseline BP (SD, mmHg)		Final SBP (SD, mmHg)		Final DBP (SD, mmHg)		Outcome (HR or RR and CI 95%)				
	SBP	DBP	Intervention	Control	Intervention	Control	Stroke	AMI	HF	CV mortality	All-cause mortality
140 to < 150 mmHg											
HOT [14] (2000)	175 (15)	105.3 (3.9)	143 (15)	147 (16)	83 (8)	80 (7)	0.85 (nd)^b^	0.72 (nd)^b^	nd	1.05 (nd)^b^	0.98 (nd)^b^
130 to < 140 versus ≥ 140 mmHg											
EWPHE [30] (1989)	182 (nd)	101 (nd)	134 (nd)	152 (nd)	84 (nd)	88 (nd)	nd	nd	nd	2.49 (1.34–4.57)	2.00 (1.28–3.09)
JATOS [10] (2008)	171.5 (9.7)	89.1 (9.5)	135.9 (11.7)	145.6 (11.1)	74.8 (9.1)	78.1 (8.9)	1.08 (0.74–1.56)	0.99 (0.33–2.80)	1.16 (0.41–3.12)	1.28 (0.48–3.38)	1.26 (0.85–1.85)
VALISH [11] (2010)	165.5 (7.9)	81.4 (6.7)	136.6 (13.3)	142 (12.5)	74.8 (8.8)	76.5 (8.9)	0.68 (0.36–1.29)	1.23 (0.33–4.56)	nd	0.97 (0.42–2.25)	0.78 (0.46–1.33)
ADVANCE [28] (2010) 65–74 yo	146 (22)	80 (11)	135 (10.6)	140.5 (15.7)	77.8 (10.6)	74 (5.1)	nd	nd	nd	0.86 (0.67–1.10)	0.84 (0.70–1.00)
ADVANCE [28] (2010) ≥ 75 yo	151 (22)	78 (11)	137 (11)	143.9 (18.2)	75.7 (8.8)	72(6.8)	nd	nd	nd	0.65 (0.44–0.98)	0.90 (0.68–1.19)
FEVER [21] (2011)	154.3 (12)^a^	92.5 (9.6)	139.7 (nd)	145.5 (nd)	81.2 (nd)	83.6 (nd)	0.56 (0.41–0.74)	nd	nd	0.51 (0.33–0.81)	0.64 (0.45–0.91)
Wei et al. [24] (2013)	159.54 (16.4)	84.2 (9.5)	135.7 (9)	149.7 (11)	76.2 (6.1)	82.1 (7.5)	0.57 (0.34–0.94)	1.00 (0.42–2.33)	0.37 (0.15–0.91)	0.49 (0.31–0.77)	0.58 (0.43–0.78)
INVEST [27] (2014)	165.4 (13.8)	90.2 (11.1)	135.8 (nd)	145 (nd)	nd	nd	0.52 (0.35–0.79)	0.83 (0.62–1.11)	0.93 (0.63–1.36)^b^	0.74 (0.56–0.99)	0.97 (0.80–1.16)
< 130 versus ≥ 130 mmHg											
LIFE [22] (2012)	176.3 (13.2) ^a^	98 (9) ^a^	120 (nd)	151 (nd)	nd	nd	nd	nd	nd	nd	1.28 (1.0–1.64)
SPS3 [12] (2015)	144.4 (20)	nd	125.2 (15.8)	137.1 (14.6)	nd	nd	1.01 (0.59–1.73)^b^	0.77 (0.23–2.52)^b^	nd	0.39 (0.16–0.92)	0.83 (0.53–1.29)^b^
SPRINT [13] (2016)	141.6 (15.7)	71.2 (11.0)	123.4 (8.3)	134.8 (8.3)	62.0 (5.5)	67.2 (6.4)	0.72 (0.43–1.21)	0.69 (0.45–1.05)	0.62 (0.40–0.95)	0.60 (0.33–1.09)	0.67 (0.49–0.91)

*BP* Blood pressure, *SD* Standard deviation, *SBP* Systolic blood pressure, *DBP* Diastolic blood pressure, *HR* Hazard ratio, *RR* Relative risk; *CI 95%* Confidence interval 95%, *AMI* Acute myocardial infarction, *HF* Heart failure, *CV* Mortality, cardiovascular mortality; nd, not described. ^a^outcome from a subanalysis or post-hoc analyses that was not adjusted. ^b^ data retrieved from the whole population

### Cardiovascular outcomes and mortality for achieving SBP levels 130 to <140 versus ≥140 mmHg

Three RCTs [10, 11, 24] and 3 p-h/sa of RCTs with moderate or low risk of bias [21, 27, 28], including 24,547 participants, compared cardiovascular and mortality outcomes in older people that achieved SBP levels 130 to <140 with those that achieved SBP levels ≥140 mmHg. In the meta-analysis of RCTs comparing achieved SBP levels of 130 to <140 mmHg with ≥140 mmHg, the lower levels were not associated with reductions in the risk of stroke, cardiovascular mortality or all-cause mortality (Fig. 2). However, in the meta-analysis of p-h/sa of RCTs**,** SBP levels 130 to <140 mmHg were associated with a 28% risk reduction of cardiovascular mortality and a 14% reduction of all-cause mortality (Fig. 2).

There was no reduction in the risk of AMI associated with the 130 to <140 mmHg levels on pooled analysis of RCTs (Fig. 2) or on the report from one p-h/sa of RCTs (Table 2) [27]. For the HF outcome, it was not possible to combine results neither from trials nor from p-h/sa of RCTs studies because there were less than 3 observations on the outcome for both study designs. Nevertheless, all studies that reported results on the outcome found no risk reduction in HF associated with the SBP levels of 130–140 mmHg (Table 2) [10, 24, 27].

### Cardiovascular and mortality outcomes for achieving SBP levels within the 140 to <150 mmHg range

One p-h/sa of RCT compared cardiovascular and mortality outcomes in older people that achieved a mean SBP of 143 mmHg with those that achieved 147 mmHg [14]. The authors found that the lower level was associated with mean reductions of 15% in the risk of stroke, of 28% in the risk of AMI and of 2% in all-cause mortality, but also with a 5% increase risk in cardiovascular mortality [14].

### Cardiovascular and mortality outcomes for achieving levels <130 versus ≥130 mmHg

Three studies, including 8834 participants, compared the SBP levels <130 mmHg with ≥130 mmHg. One study was a RCT [13] and two were p-h/sa of RCTs [12, 22]. Therefore, these studies did not fulfil our criteria for combining their results. The studies that reported on stroke and AMI outcomes found no reductions associated with the lower SBP levels (Table 2) [12, 13]. One study that analysed the HF outcome found a 38% risk reduction associated with achieving levels <130 mmHg [13]. For the cardiovascular mortality outcome, there was a tendency towards lower risk associated with achieving levels <130 mmHg in one trial [13], and a 61% risk reduction in a post-hoc analysis [12]. For the all-cause mortality outcome, data were divergent among the 3 studies. While there was no risk reduction detected in one post-hoc analysis [12], another post-hoc analysis reported a 28% increased risk [22], whereas the RCT found a 32% lower risk associated with levels <130 mmHg [13].

### Adverse events

Although one of the studies reported an increased risk of orthostatic hypotension associated with achieving SBP levels 130 to <140 when compared to ≥140 mmHg [28], there was no report of consequent increased risk of fractures [28]. Furthermore, SBP levels130 to <140 mmHg were not associated with a higher risk of kidney failure or of other serious adverse events (Table 2e from Additional file 1).

There were no comparisons of adverse events incidence available for the study in which SBP levels in both intervention and control groups were within the 140 to <150 mmHg range. Studies comparing SBP levels <130 with ≥130 mmHg found no increased risk of fractures (Table 2e from Additional file 1) [12, 13]. There was, however, a higher incidence of emergency visits due to acute renal failure associated with the SBP levels <130 mmHg (RR 1.43, CI 95% 1.02–1.99) [31].

### Sensitivity analysis and publication bias

In the meta-analyses that compared achieved SBP 130 to <140 mmHg with ≥140 mmHg, heterogeneity ranged from low (0.0% in pooled results of RCTs on AMI outcome) to high (79.3% in the analysis RCTs on all-cause mortality) (Fig. 2). Due to the limited number of studies, it was not possible to explore the factors contributing to the observed heterogeneity. In the sensitivity analysis using the fixed effect model, we found a significant reduction in the risk of cardiovascular mortality and all-cause mortality associated with the 130 to <140 mmHg level in the combined results of RCTs (Fig. 2). Other sensitivity analyses yielded results that were not substantially different (Fig. 1e from Additional file 1).

Visual inspection of Begg funnel plots for assessment of publication bias revealed symmetrical plots and Egger test estimates were non-significant (*p* > 0.05) for all analyses (Fig. 2e from Additional file 1).

## Discussion

In this systematic review, we summarized evidence on achieving SBP levels <150 mmHg in subjects aged ≥60 years old. There was suggestive evidence that achieving SBP levels of 130 to <140 mmHg is associated with reductions in the risk of cardiovascular mortality and all-cause mortality without a subsequent increased risk of serious adverse events. For SBP levels <130 mmHg, data on cardiovascular and all-cause mortality prevention are conflicting, and an increased risk of emergency visits due to acute kidney failure has been reported [13, 31]. Limited information on SBP levels within the 140 to <150 mmHg range impaired conclusions regarding these levels.

In our study, the lower risk of cardiovascular and all-cause mortality associated with achieving SBP levels of 130 to <140 mmHg found in the meta-analysis of p-h/sa of RCTs was not supported by the meta-analysis of RCTs. These divergent results may be a consequence of differences between studies regarding baseline cardiovascular risk profile of the included participants. The studies that found no benefits [10, 11] associated with the 130 to <140 mmHg levels included participants that had a lower baseline cardiovascular risk than those included in the study that found benefits [24]. Therefore, the absence of improvements associated with achieving SBP 130 to <140 mmHg found in our meta-analysis of RCTs could be partially explained by the lower cardiovascular risk profile of the participants included in these trials. Furthermore, there was a high proportion of participants with diabetes or previous coronary heart disease among those included in the meta-analyses of p-h/sa of RCTs. These findings endorse the possibility that the benefits of achieving 130 to <140 mmHg SBP levels might be restricted to older people with a high cardiovascular risk profile. Another possible reason for the diverse results is the difference between studies regarding their number of events. The two RCTs that found no benefits of lowering SBP into levels 130 to <140 mmHg had a low number of strokes, AMI, cardiovascular mortality and all-cause mortality [10, 11], which may have impaired the power of the studies to detect a possible benefit associated with the level. Therefore, the absence of reduction in the risk of cardiovascular events and all-cause mortality associated with the achieved SBP 130 to <140 mmHg found in our meta-analysis of RCTs may also have been a consequence of the low power of the trials included in the analyses [10, 11].

The paucity of data on SBP levels within the range 140 to <150 mmHg impaired analyses concerning these levels. Nevertheless, current evidence suggests that the SBP level of 143 mmHg might be associated with a reduction in the risk of stroke and AMI when compared to the SBP level of 147 mmHg. Studies reporting on SBP levels <130 mmHg yielded conflicting results. While one RCT found a reduction in all-cause mortality [13], one post-hoc analysis reported no risk reduction [12] and another study described an increased risk associated with the level [22]. Similar to the levels 130 to <140 mmHg, these discrepancies might be explained by differences between studies regarding baseline cardiovascular risk profile of the included participants. The post-hoc analysis that found a higher risk of all-cause mortality associated with SBP levels <130 mmHg [22] had included participants with lower baseline cardiovascular risk when compared to the trial that found benefits [13]. Therefore, the benefits associated with achieving SBP levels <130 mmHg might be restricted to older people with a high cardiovascular risk profile. These assumptions are further supported by recent findings of HOPE-3 trial [32]. In this RCT, the authors found no benefits of lowering the SBP into levels <130 mmHg on the composite outcomes of stroke, AMI or cardiovascular death in older participants with intermediate baseline cardiovascular risk [32].

In this meta-analysis, we provide data stratified according with the range of achieved SBP levels and study design. In a previous meta-analysis, Xie et al. found that more intensive BP control reduced the risk of cardiovascular events when compared to less intensive control in participants aged 62 years old or more [33]. However, the authors did not provide clear data on which achieved BP levels were being analysed in the more intensive and less intensive groups [33]. Bavishi et al. meta-analysis of RCTs found that SBP levels <140 mmHg decreased major adverse cardiovascular events (MACE) when compared to attaining higher levels [34]. However, analyses stratified according with achieved SBP ranges were not provided, therefore, the results of the study do not clarify whether the decreased MACE was actually associated with the <130 mmHg SBP levels [34]. Contradicting these results, Weiss et al. found no benefits of lowering SBP into levels <140 mmHg when compared with attaining higher levels [35]. The discrepancy found in their results might be due to the inclusion of participants receiving only placebo for hypertension treatment in the control group of studies analysing achieved SBP levels >140 mmHg [35]. We critically appraised the literature following an a priori designed protocol with clearly defined inclusion and exclusion criteria and providing mean BP levels achieved in both intervention and control groups.

Main limitations of the present review are the relatively small number of eligible studies, low number of incident events and small sample size of some of the included studies. Because of these limitations, it was not possible to provide stratified analyses for older ages (e.g. 70 or 80 years old), which would be interesting since the 60 years old cut-off for definition of older people chosen in this study might be considered too low. However, the results from our sensitivity analyses on studies including participants aged ≥65 years old were similar to the results of the whole study. Furthermore, the mean age of the participants included in our study was 72 years old, which meets more strict criteria for definition of the older population [8]. The small number of studies impaired our ability to find which cardiovascular outcome might be responsible for the reduction in cardiovascular death, and to provide statistical analyses of potential sources for the heterogeneity found in our study. Differences between studies regarding sample sizes, follow-up period, baseline cardiovascular risk profile, ethnicity and socioeconomic factors might be explanations for the heterogeneity found in our meta-analyses. Since older people are especially susceptible to serious adverse events associated with antihypertensive drug treatment, a risk-benefit analysis could provide more information to support the indication of pharmacotherapy in this population. Our attempt to provide such risk-benefit analysis was precluded by the considerable differences between studies regarding the definition of serious adverse events.

Future trials providing data on outcomes stratified by different cardiovascular risk profiles could further identify which profile benefits best from each SBP level. Moreover, trials including a larger number of participants and with a longer follow-up period could correct power issues observed in previous trials and provide important information regarding serious adverse events. Three trials are currently recruiting participants to analyse the impact of different SBP levels <150 mmHg in cardiovascular outcomes [16, 18, 36]. Two will evaluate participants with previous stroke [16, 18] and one will provide information for the general population [36]. These studies may help clarify whether older people with different cardiovascular risk profile might benefit from different SBP levels.

## Conclusions

In this review, we found there is suggestive evidence that achieving SBP levels 130 to <140 mmHg is associated with reductions in the risk of cardiovascular and all-cause mortality without a subsequent increased risk of serious adverse events among subjects aged ≥60 years old. Therefore, we showed that the evidence favoring this level is stronger than experts’ opinion. Nevertheless, future trials including a larger number of participants with different cardiovascular risk profiles and longer follow-up periods are required to confirm these findings, to clarify which cardiovascular events might be prevented when the level 130 to <140 mmHg is achieved and to provide additional evidence regarding the <130 and 140 to <150 mmHg SBP levels.

## Additional files

Additional file 1: Supplementary information on Search strategy, Supplementary information on Data Synthesis, Fig. 1e (Sensitivity analyses); Fig. 2e (publication bias), Table 1e (Location and adjustments done in each study), Table 2e (Adverse events in different achieved systolic blood pressure levels for the elderly), Table 3e (Quality assessment for RCTs); Table 4e (Quality assessment for subanalyses and post-hoc analyses of RCTs). (DOCX 237 kb)

Additional file 2 Check-list for evaluation of quality of meta-analyses. (DOCX 17 kb)

## Abbreviations

95% CI: 95% confidence interval
AMI: Acute myocardial infarction
DBP: Diastolic blood pressure
Hf: Heart failure
MACE: Major adverse cardiovascular events
p-h/sa of RCTs: Post-hoc analyses and subanalyses of randomized controlled trials
RCTs: Randomized controlled trials
RR: Relative risk
SBP: systolic blood pressure
