# Supplementary material for: Achieved systolic blood pressure in older people: a systematic review and meta-analysis
Source: BMC Geriatr. 2017 Dec 5;17:279. doi: 10.1186/s12877-017-0672-4 (PMC5717809; doi:10.1186/s12877-017-0672-4)
Supplement: Supplementary file 1 — Supplementary information on Search strategy, Supplementary information on Data Synthesis, Fig. 1e (Sensitivity analyses); Fig. 2e (publication bias), Table 1e (Location and adjustments done in each study), Table 2e (Adverse events in different achieved systolic blood pressure levels for the elderly), Table 3e (Quality assessment for RCTs); Table 4e (Quality assessment for subanalyses and post-hoc analyses of RCTs). (DOCX 237 kb) [file 12877_2017_672_MOESM1_ESM.docx]

**Additional file 1**

**Supplementary information on Search strategy**

Relevant studies published from their inception until July 8^th^ 2016 (date last searched), were identified through electronic searches not limited to the English Language using EMBASE, MEDLINE, Web-of-Science, Cochrane, Google Scholar, PubMed, Lilacs and Scielo. Electronic searches were supplemented by scanning reference lists of articles identified for all relevant studies (including review articles), by hand searching of relevant journals and by correspondence with study investigators and specialists.

(i) MEDLINE strategy to identify relevant exposures:

(exp "antihypertensive agents"/ OR exp hypertension/dt OR (antihypertens* OR (anti ADJ hypertens*) OR hypotensiv* OR (("blood pressure" OR hypertens*) ADJ3 (lower* OR treat* OR therap* OR drug* OR agent*)) OR atenolol OR hydrochlorothiazide OR amlodipine OR furosemide).ab,ti.)

(ii) MEDLINE strategy to identify relevant outcomes:

AND (exp "stroke"/ OR exp "Cerebral Arterial Diseases"/ OR exp "Myocardial Infarction"/ OR mortality/ OR mortality.xs. OR ((cerebrovasc* ADJ3 accident*) OR cva OR stroke OR infarction OR mortalit* OR (cardiovasc* ADJ3 event*)).ab,ti.)

(iii) MEDLINE strategy to identify relevant population:

("middle aged"/ OR exp aged/ OR aging/ OR ("middle age" OR aged OR aging OR older OR old OR ageing OR elderl* OR senior*).ab,ti.) NOT (Animals/ NOT Humans/ )

(iv) MEDLINE strategy to identify relevant study design:

(Clinical Trial.pt. OR (randomized OR placebo OR randomly OR trial OR groups).ab,ti.) NOT (Review OR congresses OR Note OR Editorial OR Letter).pt.

Parts i, ii, iii and iv were combined using ‘AND’ to search the MEDLINE. Each part was specifically translated for searching alternative databases.

**Supplementary information on Data Synthesis**

When achieved SBP was reported as a categorical variable, we assumed it to have a normal distribution and considered the mean as the achieved SBP [30]. In the case when the range was not described, we imputed the achieved SBP as the weighted mean achieved SBP for all studies [28]. For studies that compared two or more intervention groups with one control group, we chose the intervention group in which the mean achieved SBP was more similar to the overall mean [28]. Studies reporting ≥ 2 independent intervention-control groups were considered as independent reports [30]. In this case, if the number of adverse events was too small (i.e. < 2% absolute incidence), we combined the results for the intervention groups and for the control groups to provide a more precise estimative [28]. We calculated mean weighted achieved systolic and diastolic for intervention and control groups. When standard deviations were not reported, they were calculated whenever there was enough information available [25]. Trials in which information on the outcomes for the elderly population was considered as a pre-specified analysis were considered as RCTs [13].

Heterogeneity was distinguished as low (I^2^ ≤25%), moderate (I^2^ >25% and <75%) or high (I^2^ ≥75%).


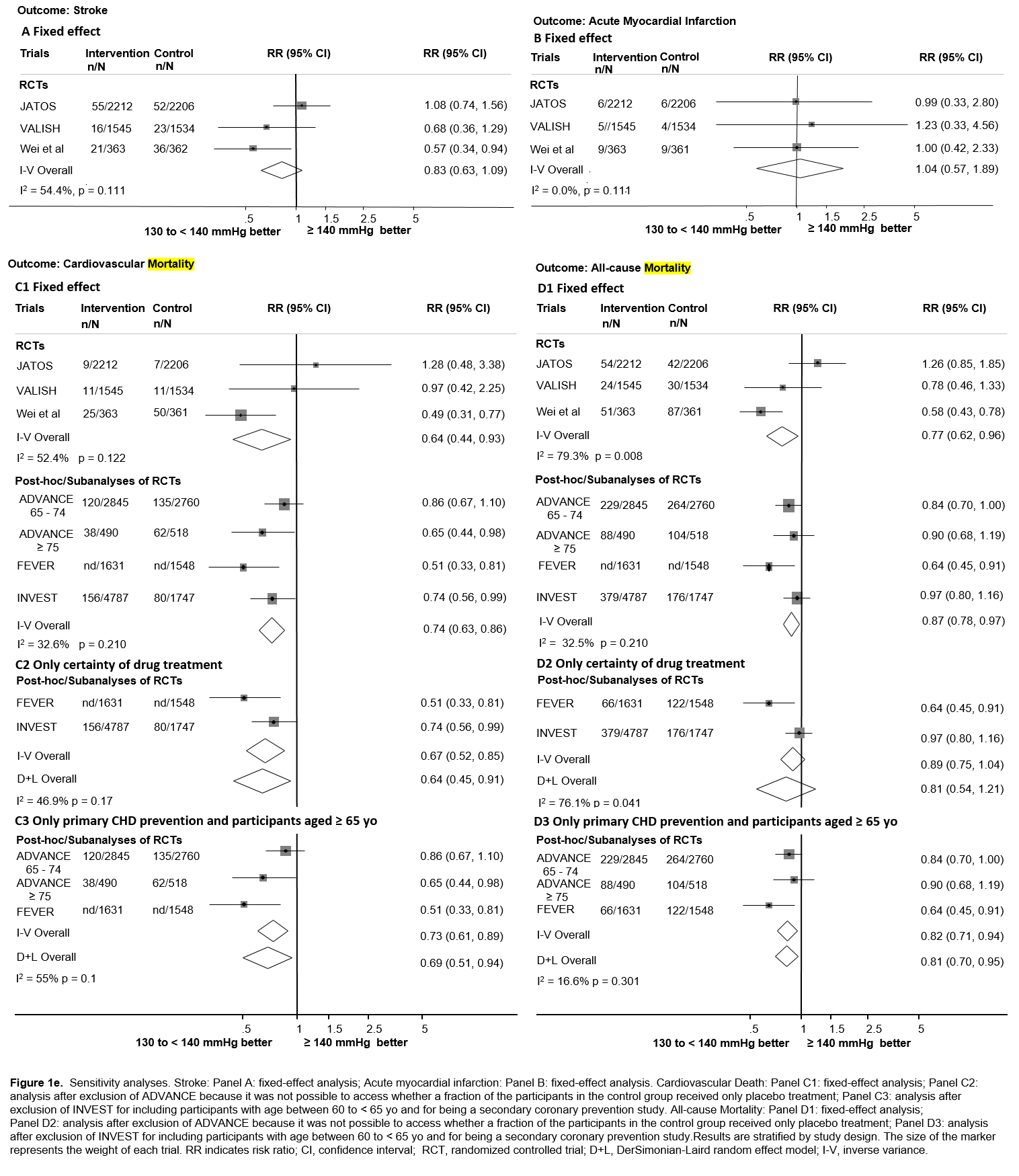


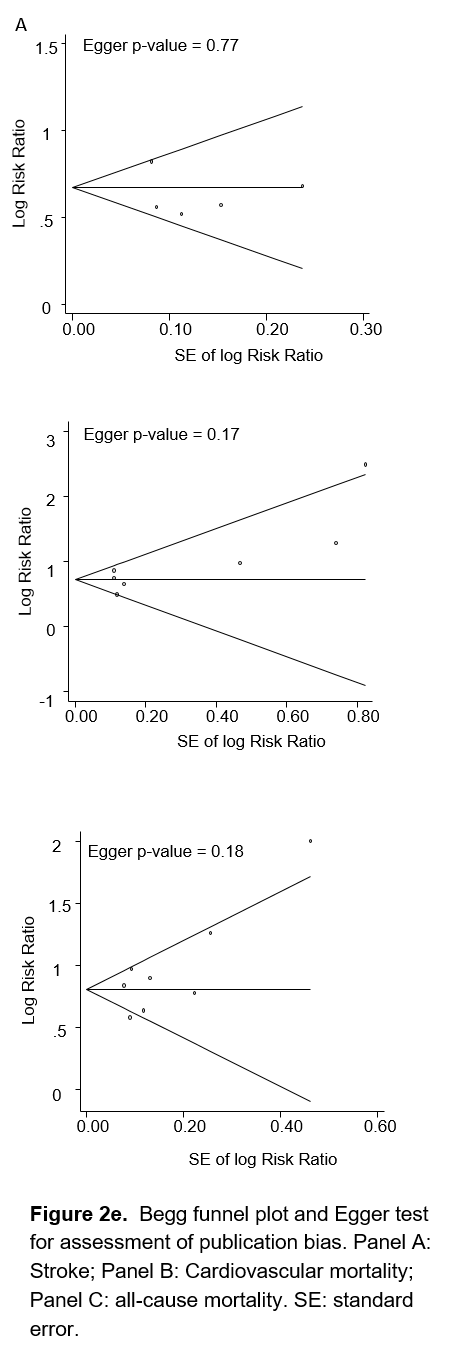


| **Table 1e**. Location and adjustments done in each study | | |
| --- | --- | --- |
| **Study name (year)** | **Location** | **Adjustment** |
| EWPHE [30] (1989) | Europe | Sex and age |
| HOT [14]  (2000) | Europe, North America, China, Israel, Argentina, Southest Asia, South Korea | Not done |
| JATOS [10] (2008) | Japan | na |
| VALISH [11] (2010) | Japan | Sex, age, BMI, smoking, dyslipidemia, diabetes mellitus and antihypertensive agents used before enrollment - since this was a RCT for elderly. |
| ADVANCE [28] (2010) | Canada, Europe, Asia, Australasia | Multivariate-adjusted analyses were conducted by using Cox model including potential confounding baseline covariates, including sex, duration of diabetes, urinary albumin-creatinine ratio, eGFR, SBP, history of currently treated hypertension, history of macrovascular disease, HbA1c, low-density lipoprotein cholesterol, high-density lipoprotein cholesterol, triglycerides, BMI, ECG abnormalities, current smoking, current drinking, randomized BP treatment and randomized glucose treatment. |
| FEVER [21] (2011) | China | Baseline SBP, previous cardiovascular disease, diabetes mellitus, gender, smoking, cholesterol, left ventricular hypertrophy, isolated systolic hypertension. |
| LIFE [22] (2012) | USA, Europe | Treatment with losartan vs atenolol, age, sex, race, diabetes, history of ischemic heart disease, myocardial infarction, stroke, peripheral vascular disease, heart failure, current smoking, prior antihypertensive treatment, baseline Sokolow-Lyon voltage, BMI, albumin/creatinine ratio, total an HDL cholesterol, serum glucose and creatinine and baseline systolic pressure entered as standard covariates, and baseline and in-treatment diastolic blood pressure, Cornell voltage-duration product left ventricular hypertrophy and heart rate entered as time-varying covariates. |
| Wei *et al* [24] (2013) | China | na |
| INVEST [27] (2014) | USA, Canada Central America, Europe, Australasia | Age, sex, race, BMI, SBP, DBP, HR, Verapamil SR strategy, diabetes, hypercholesterolemia, angina, CABG or PCI, stroke or TIA, LVH, unstable angina, heart failure, peripheral vascular disease, renal impairment, cancer, well-being, use of statins, nitrate, antiplatelet agents, NSAIDs, potassium supplement, antidiabetic medication and hormone replacement |
| SPS3 [12] (2015) | USA, Canada, Spain, South America | Done only for cardiovascular mortality outcome and adjusted for: sex, race, ethnicity, diabetes mellitus, hypertension, ischemic heart disease, hyperlipidemia and smoking status. |
| SPRINT [13] (2016) | USA and Puerto Rico | na |
| Na, not applicable; BMI, body mass index; RCT, randomized controlled trial; eGFR, estimated glomerular filtration rate; SBP, systolic blood pressure; ECG, electrocardiogram; BP, blood pressure; DBP, diastolic blood pressure; HR, heart rate; CABG, coronary artery bypass graft; PCI, percutaneous coronary intervention; TIA, transitory ischemic attack; LVH, left ventricular hypertrophy; NSAIDs, non-steroidal anti-inflammatory drugs. | | |

| **Table 2e.** Adverse events in different achieved systolic blood pressure levels for the elderly | | | |
| --- | --- | --- | --- |
| **Study name (year)** | **Serious Adverse Events, Kidney failure or fractures n(%) intervention *versus* n(%) control** | **Discontinuation due to adverse events**  **n(%) intervention *versus* n(%) control** | **Any adverse events n(%) intervention *versus* n(%) control** |
| **140 to < 150 mmHg** | |  |  |
| HOT [14]  (2000) | nd | nd | For all participants ≥ 65 years old:  Peripheral edema: 898 (15)  Cough: 221 (3.7)  Dizziness: 191 (3.2)  Hypotension: 65 (1.1)  Dyspepsia: 83 (1.4) |
| **130 to < 140 *versus* ≥ 140 mmHg** | |  |  |
| EWPHE [30] (1989) | nd | nd | nd |
| JATOS [10] (2008) | Kidney failure €: 8 (0.36) vs 9 (0.40), p = 0.8 | 36 (1.6) vs 36 (1.6), p = 0.99 | Gastrointestinal signs and symptoms or abnormal laboratory findings: 550 (24) vs 548 (24), p = 0.99 |
| VALISH [11]  (2010) | Kidney failure₠: 5 (0.32) vs 2 (0.13) p = 0.26₠  Any serious adverse events: 86 (5.6) vs 67 (4.4), p = 0.126 | 29 (1.9) vs 18 (1.2), p = 0.11 | Any adverse events: 281(18.2) vs (17.9), and most were gastrointestinal or respiratory, p = 851 |
| ADVANCE [28] (2010)  65 - 74 yo | Discontinuation due to serious adverse events: 35 (1.2) vs 35 (1.3), p = ns^a^  Renal events: 598 (21.1) vs 729 (26.3), p = significant^b^  Leg fracture: 2 (0.07%) vs 1 (0.03%), p = ns | 573 (20.2) vs 546 (19.7), p = ns | Cough: 107 (3.8) vs 41 (1.5), p = significant  Discontinuation due to hypotension or dizziness: 36 (1.3) vs 13 (0.5), p = significant  Discontinuation due to hyperkalemia: 4 (0.1) vs 6 (0.2), p = ns |
| ADVANCE [28] (2010)  ≥ 75 yo | Discontinuation due to serious adverse events^a^: 10 (2.1) vs 12 (2.3), p = ns  Renal events: 116 (24.0) vs 150 (28.6), p = significant^b^  Leg fracture: 1 (0.2) vs 0 (0.0), p = ns | 144 (29.8) vs 158 (30.1), p = ns | Cough: 21 (4.3) vs 8 (1.5), p = significant  Discontinuation due to hypotension or dizziness: 11 (2.3) vs 2 (0.4) p = significant  Discontinuation due to hyperkalemia: 1 (0.2) vs 1 (0.2), p = ns |
| FEVER [21]  (2011) | nd | nd | nd |
| Wei *et al* [24] (2013) | Femoral fracture: 3 (0.8) vs 5 (1.3) , p = 0.716  Death by uremia: 1 (0.3) vs 4 (1.1), p = 0.36 | nd | nd |
| INVEST [27]  (2014) | Renal failure: 35 (0.7) vs 14 (0.8), p = nd | nd | Peripheral edema: 31 (0.6) vs 31 (1.8), p = nd  Cough: 153 (3.2) vs 71 (4.1), p = nd  Dizziness: 108 (2.3) vs 68 (3.9), p = nd  Hyperkalemia: 4 (0.1) vs 4 (0.2), p = nd  Hypokalemia: 11 (0.2) vs 5 (0.3), p = nd  Symptomatic bradycardia: 83 (1.7) vs 46 (2.6), p = nd  Wheezing: 28 (0.6) vs 14 (0.8), p = nd |
| **< 130 *versus* ≥ 130 mmHg** | |  |  |
| LIFE [22]  (2012) | nd | nd | nd |
| SPS3 [12]  (2015) | Fall with injury secondary to hypotension 1 (0.11) vs 0 (0), p > 0.99 | nd | Postural hypotension 131 (53) vs 159 (65), p = 0.008  Orthostatic syncope 2 (0.21) vs 1 (0.11), p = 0.58 |
| SPRINT [13, 31] (2016) | An event that was fatal or life threatening resulting in significant or persistent disability, requiring or prolonging a hospitalization or was an important medical event that the investigator judged to be a significant hazard or harm to the participant that may have required medical or surgical intervention to prevent one of the other events listed above:  640 (48.6) *vs* 638 (48.4) p = 0.93  Acute Kidney Injury or Acute Renal Failure 78 (5.9) vs 55 (4.2) p = 0.05 ¢ | nd | Hypotension 36 (2.7) vs 24 (1.8), p = 0.13  Orthostatic hypotension 277(21) vs 288 (21.8) ¥  Orthostatic hypotension with dizziness 25 (1.9) vs 17 (1.3)  Syncope 46 (3.5) vs 37 (2.8), p = 0.33  Bradycardia 41 (3.1) vs 43 (3.3), p = 0.79  Electrolyte abnormality 58 (4.4) vs 41 (3.1), p = 0.10  Injurious fall 70 (5.3) vs 79 (6.0), p = 0.42 £ |

Nd, not described; na, not applicable; ns, non-significant; * defined as one or more of: sodium ≤ 130 mmol/l, potassium ≥ 5.5 mmol/l or potassium ≤ 3 mmol/l; § defined as creatinine ≥ 170 μmol/l; £ defined as a fall that resulted in evaluation in an emergency department or resulted in hospitalization; ¢: if the diagnosis was listed in the hospital discharge summary and was felt to be one of the top 3 reasons for admission or continued hospitalization; ¥ defined as drop in systolic BP ≥ 20 mmHg or drop in diastolic ≥ 10 mmHg 1 minute after standing - participants were asked if they felt dizzy at the tie the orthostatic measure was taken; ₠ doubling of serum creatinine to a level > 2.0mg/100ml or introduction of dialysis; € acute or chronic renal failure, doubling of serum creatinine concentration to a value of 1.5 mg/dL or higher; ^a^ discontinuation of study treatment due to any serious adverse events (*e.g*., suspected adverse drug reactions, macro or microvascular events or heart failure); ^b^ development of micro or macroalbuminuria, doubling of serum creatinine to a level of at least 200mmol/l, need for renal replacement therapy and death due to renal disease.

| **Table 3e.** Quality assessment for RCTs (all outcomes). | | | | | | | |
| --- | --- | --- | --- | --- | --- | --- | --- |
| **Study name** | **Sequence generation** | **Allocation concealment** | **Blinding of participants and personnel** | **Blinding of assessment** | **Missing data** | **Selective reporting** | **Other bias** |
| Wei *et al* [24] | + | + | + | + | + | + | + + |
| JATOS [10] | + | + | + | + | + | + | + + |
| VALISH [11] | + + | + + | + | + | + + | + | + + |
| SPRINT[13] | + | + | + | + | + | + | + |

RCTs, randomized controlled trials. +: low risk of bias; + +: unclear risk of bias; + + +: high risk of bias.

| **Table 4e.** Quality assessment for subanalyses and post-hoc analyses of RCTs for each outcome. | | | | | | | | |  |
| --- | --- | --- | --- | --- | --- | --- | --- | --- | --- |
| **Quality assessment for subanalyses and post-hoc analyses of RCTs for stroke outcome** | | | | | | | | |  |
| **Study name** | **Exposed cohort representativeness** | **Non-exposed cohort selection** | **Ascertainment of exposure** | **Outcome of interest not present at start** | **Comparability** | **Assessment of outcome** | **Long enough FU** | **Adequacy of FU** | **Bias risk classification** |
| HOT [14] | * | * | * | * | 0 | * | * | * | Moderate |
| FEVER [21] | * | * | * | * | * | * | * | * | Moderate |
| INVEST [27] | * | * | * | * | ** | * | 0 | * | Moderate |
| SPS3 [12] | * | * | * | * | * | * | 0 | * | Moderate |
| **Quality assessment for subanalyses and post-hoc analyses of RCTs for AMI outcome** | | | | | | | | |  |
| HOT [14] | * | * | * | * | 0 | * | * | * | Moderate |
| INVEST [27] | * | * | * | * | ** | * | 0 | * | Moderate |
| SPS3 [12] | * | * | * | * | * | * | 0 | * | Moderate |
| **Quality assessment for the post-hoc analysis of RCTs for HF outcome** | | | | | | | | |  |
| INVEST [27] | * | * | * | * | ** | * | 0 | * | Moderate |
| **Quality assessment for subanalyses and post-hoc analyses of RCTs for cardiovascular mortality outcome** | | | | | | | | |  |
| HOT [14] | * | * | * | * | 0 | * | * | * | Moderate |
| EWPHE [30] | * | * | * | * | * | * | 0 | 0 | High |
| ADVANCE [28] | * | 0 | * | * | ** | * | * | * | Moderate |
| FEVER [21] | * | * | * | * | * | * | * | * | Moderate |
| INVEST [27] | * | * | * | * | ** | * | 0 | * | Moderate |
| SPS3 [12] | * | * | * | * | ** | * | 0 | * | Moderate |
| **Quality assessment for subanalyses and post-hoc analyses of RCTs for all-cause mortality** | | | | | | | | |  |
| HOT [14] | * | * | * | * | 0 | * | * | * | Moderate |
| EWPHE [30] | * | * | * | * | * | * | 0 | 0 | High |
| ADVANCE [28] | * | 0 | * | * | ** | * | * | * | Moderate |
| FEVER [21] | * | * | * | * | * | * | * | * | Moderate |
| INVEST [27] | * | * | * | * | ** | * | 0 | * | Moderate |
| LIFE [22] | * | * | * | * | * | * | * | * | Moderate |
| SPS3 [12] | * | * | * | * | * | * | 0 | * | Moderate |

RCTs, randomized controlled trials; FU, follow up; AMI, acute myocardial infarction; HF, heart failure. Low risk of bias: 9 stars; Moderate risk of bias: 7 or 8 stars; High risk of bias: ≤ 6 stars.
